# Corticosteroid Management of Coronavirus 2019 (COVID-19) in Patients with Bilateral Adrenalectomy

**DOI:** 10.1155/2021/5856600

**Published:** 2021-10-13

**Authors:** William Lim, Frederick Lim

**Affiliations:** ^1^Department of Internal Medicine, Richmond University Medical Center, Staten Island, NY, USA; ^2^Department of Endocrinology/Internal Medicine, Prisma Health, Columbia, SC, USA

## Abstract

Since the World Health Organization (WHO) announced coronavirus 2019 (COVID-19) as a pandemic in March 2020, it has been wreaking havoc across countries, affecting people's lives. Corticosteroids have proven to provide a mortality benefit in patients with COVID-19. Although dexamethasone is the most commonly used glucocorticoid and have shown to have mortality benefit in COVID-19 patients, it cannot be used in patients with adrenal insufficiency due to its lack of mineralocorticoid activity. Herein, we discuss a case of challenging corticosteroid management in a patient with COVID-19 complicated by her medical history of bilateral adrenalectomy.

## 1. Introduction

Since the end of 2019, coronavirus disease 2019 (COVID-19) has rapidly spread across the whole world, resulting in a pandemic. With over 100 million cases and 3 million deaths, COVID-19 is wreaking havoc across the globe. Dexamethasone is the most commonly used glucocorticoid that has shown a mortality benefit in patients with COVID-19 [1–3]. We herein report a case of steroid management in a patient with a medical history of bilateral adrenalectomy presenting to the emergency room with COVID-19. To the best of our knowledge, there has been no report of COVID-19 associated with the medical history of bilateral adrenalectomy.

## 2. Case Presentation

A 58-year-old female with past medical history of multiple endocrine neoplasia type 2 (MEN2) status after thyroidectomy and bilateral adrenalectomy and right breast lobectomy, carpal tunnel syndrome, anxiety, depression, and asthma presented to the emergency department (ED) after testing positive for COVID-19. The patient stated that she was in her usual state of health until 3 days prior to admission when she started having fever, persistent cough with whitish phlegm, mild shortness of breath, and watery diarrhea together with generalized body aches, headache, and excessive sweating.

**Figure 1:**
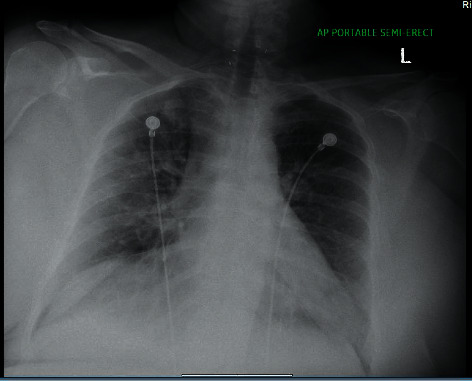
Chest X-ray showing bilateral infiltrates.

She decided to take a COVID-19 test which came back positive which prompted her to come to the ED. Her home medications included hydrocortisone 20 mg in the morning, 10 mg in the afternoon, and 5 mg at night, levothyroxine 112 mcg two tablets in the morning, and fludrocortisone 0.1 mg tab daily. Her vital signs were as follows: temperature, 98.7°F; blood pressure, 100/48 mmHg; heart rate, 98/min; and O2 saturation, 95% on 2 litres of nasal cannula and 88% on room air. Random blood glucose level is 136 mg/dl. Chest X-ray (Figure 1) showed bilateral infiltrates. Remdesivir 200 mg one time and 100 mg for 4 more days was started intravenously (IV) for acute hypoxic respiratory failure. Hydrocortisone 100 mg IV every 8 hours for 2 doses was given while awaiting the endocrinologist to see the patient, and the patient's home medication fludrocortisone was held.

**Figure 2:**
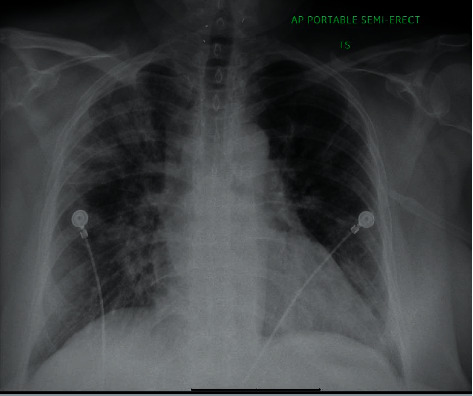
Chest X-ray showing an improvement in bilateral infiltrates.

Since the patient received another 100 mg of hydrocortisone apart from bolus dose, a decision was made to continue hydrocortisone 50 mg IV for 2 more doses: a total of 200 mg for the first 24 hours. The patient's condition improved the next day, and hydrocortisone was switched to oral hydrocortisone at the strength of double her home dose: 40 mg in the morning, 20 mg in the afternoon, and 5 mg at night. After 3 more days of double strength of hydrocortisone, patient condition improved, and repeat CXR (Figure 2) showed an improvement. The patient was discharged with his regular home dose of hydrocortisone and fludrocortisone.

## 3. Discussion

Severe acute respiratory syndrome coronavirus is an enveloped single-stranded positive-stranded RNA virus that enters the human cells by binding to its cellular receptor, angiotensin-converting enzyme 2 (ACE2) [4, 5]. Direct person-to-person transmission is the primary means of transmission. It is thought to occur mainly through close-range contact via respiratory particles [6]. The incubation period for COVID-19 is generally within 14 days following exposure [7–9]. Most of the patients present with nonspecific flu-like symptoms or hypoxic respiratory failure with severe pneumonia [9–12].

Dexamethasone is the most commonly used glucocorticoid for COVID-19 pneumonia and shown to have a mortality benefit for severely ill COVID-19 patients who are on supplemental oxygen or ventilatory support [1–3]. However, it cannot be used in patients with adrenal insufficiency due to its lack of mineralocorticoid activity. There are only limited data on the efficacy of other glucocorticoids in treatment of COVID-19 patients because these trials were stopped early [13, 14].

Hydrocortisone is the glucocorticoid with mineralocorticoid activity and preferred glucocorticoid in patients with adrenal insufficiency [15]. Hydrocortisone 100 mg IV bolus followed by 50 mg IV every 6 hours is the recommended dose for patients with adrenal crisis [16]. In the abovementioned case, the patient who is taking hydrocortisone and fludrocortisone as a maintenance home dose presented to the ED with acute hypoxic respiratory failure due to COVID-19. We used a similar regimen that was used in adrenal crisis (100 mg IV bolus and 200 mg IV in the 24 hours) and saw an improvement in patient condition.

During minor illnesses, increasing the dose of glucocorticoid to two to three times the usual daily dose for three days is recommended and known as the 3 × 3 rule [17].

Since our patient's clinical condition improved, IV hydrocortisone was switched to oral dose at a double strength. Fludrocortisone was held while the patient was receiving double strength of usual hydrocortisone dose since hydrocortisone 20 mg has an equivalent mineralocorticoid effect of fludrocortisone of 0.1 mg [15].

## 4. Conclusions

The study discussed a challenging case of corticosteroid management in a patient with COVID-19 which was complicated by her medical history of bilateral adrenalectomy. This case adds to a growing body of literature of the effectiveness of alternative glucocorticoids other than dexamethasone use in COVID-19 patients with hypoxic respiratory failure and highlights the importance of understanding doses and effects of different glucocorticoids.

## Data Availability

No data were used to support this study.

## Abbreviations

mg: Milligram
mcg: Microgram
F: Fahrenheit
mmHg: Millimeter of mercury
min: Minute.
